# The specific impact of uremic toxins upon cognitive domains: a review

**DOI:** 10.1590/2175-8239-JBN-2018-0033

**Published:** 2018-08-09

**Authors:** Álvaro de Oliveira Franco, Rodrigo Tzovenos Starosta, Matheus Roriz-Cruz

**Affiliations:** 1 Universidade Federal do Rio Grande do Sul, Faculdade de Medicina, Porto Alegre, RS, Brasil.; 2 Universidade Federal do Rio Grande do Sul, Programa de Pós-Graduação em Genética e Biologia Molecular, Porto Alegre, RS, Brasil.; 3 Universidade Federal do Rio Grande do Sul, Departamento de Medicina Interna, Porto Alegre, RS, Brasil.; 4 Hospital de Clínicas de Porto Alegre, Porto Alegre, RS, Brasil.

**Keywords:** Uremia, Cognitive Dysfunction, Knowledge, Renal Insufficiency, Chronic, Renal Dialysis, Toxins, Biological, Memory, Executive Function, Attention, Uremia, Disfunção Cognitiva, Conhecimento, Insuficiência Renal Crônica, Diálise Renal, Toxinas Biológicas, Memória, Função Executiva, Atenção

## Abstract

One of the mechanisms proposed for chronic kidney disease (CKD)-related cognitive impairment is the accumulation of uremic toxins due to the deterioration of the renal clearance function. Cognition can be categorized into five major domains according to its information processing functions: memory, attention, language, visual-spatial, and executive. We performed a review using the terms 'uric acid', 'indoxyl sulfate', '*p*-cresyl sulfate', 'homocysteine', 'interleukins' and 'parathyroid hormone'. These are the compounds that were found to be strongly associated with cognitive impairment in CKD in the literature. The 26 selected articles point towards an association between higher levels of uric acid, homocysteine, and interleukin 6 with lower cognitive performance in executive, attentional, and memory domains. We also reviewed the hemodialysis effects on cognition. Hemodialysis seems to contribute to an amelioration of CKD-related encephalopathic dysfunction, although this improvement occurs more in some cognitive domains than in others.

## RESUMO

Um dos mecanismos propostos para explicar o comprometimento cognitivo relacionado à doença renal crônica (DRC) é o acúmulo de toxinas urêmicas devido à deterioração da função de depuração renal. A cognição pode ser categorizada em cinco domínios principais de acordo com suas funções de processamento de informações: memória, atenção, linguagem, visual-espacial e executiva. Realizamos uma revisão usando os termos "ácido úrico", "indoxil sulfato", "*p*-cresil sulfato", "homocisteína", "interleucinas" e "paratormônio". Estes são os compostos que se mostraram fortemente associados ao comprometimento cognitivo na DRC na literatura. Os 26 artigos selecionados apontam para uma associação entre níveis mais elevados de ácido úrico, homocisteína e interleucina-6 com menor desempenho cognitivo nos domínios executivo, atenção e de memória. Também revisamos os efeitos da hemodiálise na cognição. A hemodiálise parece contribuir para uma melhoria da disfunção encefalopática relacionada à DRC, embora essa melhora ocorra mais em alguns domínios cognitivos do que em outros.

## INTRODUCTION

Many potentially toxic compounds build up in patients with chronic kidney disease (CKD); the biologically active ones are called uremic toxins (UTs)^1^^,^ 2. UTs comprise around 150 compounds that may cause many deleterious effects, such as systemic inflammation, cardiac failure, anemia, immune dysfunction, anorexia^2^, neurological damage, and cognitive impairment.

CKD patients have a higher risk of developing cognitive impairment (CI) related to CKD (CKD-CI) even in the earlier stages^3^^-^ 7, which affects their daily life and work capacity, and causes increased periods of hospitalization^8^. Most importantly, CKD-CI is an independent predictor of mortality in patients submitted to dialysis and is associated with an almost three times greater mortality risk in 7 years^9^. More than 70% of hemodialysis (HD) patients older or equal to 55 years have moderate-to-severe CKD-CI^10^.

CKD-CI's hypothetical mechanism can be divided into neurodegenerative^8^^,^ 11 ^-^ 13 and cerebro-vascular^14^^-^ 16 components. The former accounts for UTs' direct neurotoxicity, resulting in the alteration of the brain's redox environment, along with the promotion of central nervous system excitotoxicity through the activation of the glutamatergic pathways and the inhibition of the GABAergic ones^17^. The latter states that UTs^14^ along with systemic hemodynamic impairment related to CKD^15^ also cause a direct effect on the cerebral endothelium, resulting in oxidative stress, chronic inflammation, hypercoagulability^8^^,^ 18 ^,^ 19, and disruption of the blood-brain barrier and cellular water transport^20^^,^ 21. This corroborates the fact that CKD patients have a higher incidence of cerebral microbleeds^22^^,^ 23, silent brain infarcts, and white matter lesions (leukariosis)^10^, even when adjusted for common risk factors (e.g.: hypertension and diabetes mellitus)^24^^,^ 25. Nevertheless, the impact of specific UTs upon cognition and the exact mechanisms by which they occur are still not completely understood, despite the increasing necessity for a systematic characterization that could improve the identification and management of CKD-CI^26^.

Therefore, we reviewed literature data regarding the mechanisms by which homocysteine and uremic toxins with a higher impact on the emergence of CKD-CI.^26^ - uric acid, indoxyl sulphate, *p*-cresyl sulphate, interleukins 1-β and 6, and parathyroid hormone - can produce deleterious effects on cognition (data shown in Table 1). We conducted an analysis on the possible relationship between the main uremic toxins on one side, and the basic cognitive domains on the other (data shown in Table 2), which reveal a consistent cognitive deterioration pattern associated with CKD-CI. Finally, we also reviewed the influence of HD upon each different cognitive domain among CKD patients, identifying which domains benefit the most from this treatment.

**Table 1:** Current mechanistic data on some of the most meaningful uremic toxins, as stated by Watanabe and colleagues26 with the addition of HCy

Uric acid	Antioxidant and pro-oxidant effects, white matter atrophy, and cerebral ischemic burden. Uric acid is a major alarmin that induces pro-inflammatory cytokine expression and secretion, as well as inflammation; the underlying mechanism for these functions is the activation of the nuclear factor-κ B by toll-like receptor 4. This response was activated more in neurons than in glial cells when rat hippocampi were studied. The promotion of gliosis has also been observed. Uric acid is also associated with atherosclerosis, endothelial and cardiovascular disease burden, microvascular renal disease, glomerular hypertension, glomerulosclerosis, and renal interstitial fibrosis. 29 30 32 64 65 66
Indoxyl sulphate and *p*-cresyl sulphate	Direct neurotoxicity of indoxyl sulphate is suggested, but not proven. Indoxyl sulphate possibly causes a disruption of the brain efflux transport systems. Some of the transporters found in brain capillary endothelium are the same secretory transport molecules found in the basolateral membrane of proximal tubular cells; for instance, the organic anion transporter 3 (OAT3). Indoxyl sulphate was found to accumulate in uremic patients’ brains. Indoxyl sulphate also causes nephrotoxic renal fibrosis through the accumulation in renal tubular cells, production of free radicals, inflammation, endothelial cell dysfunction, endothelial and proximal tubular cell senescence, atherosclerosis, and the disruption of rhythmicity regulation of clock genes (rPer2). 26 67 68
Homocysteine (HCy)	HCy increases oxidative stress, DNA damage, induction of apoptosis, production of homocysteic acid, excitotoxicity (mediated by NMDA glutamate receptor activation), white matter hyperintensities, cerebrovascular disease, and brain atrophy. Hyperhomocysteinemia is linked to cerebral microvascular rarefaction and dysfunction of the methylation of DNA, proteins, and phospholipids due to the inhibition of methyltransferase. This can lead toabnormal epigenetic regulation. Superoxide and hydrogen peroxide are formed by the oxidation of homocysteine, whose increased levels could cause a reduction in glutathione peroxidase activity and antioxidant potentials. Hyperhomocysteinemia also seems to cause alterations in the monoamine neurotransmitter system through mechanisms involving the inhibition of methyltransferase reactions and changes in the cellular redox state. Involving these same mechanisms, hyperhomocysteinemia also promotes the reduction of brain-derived neurotrophic factor (BDNF) levels in cerebrospinal fluid. BDNF is a protein related to cell maintenance, plasticity, growth and death. Hyperhomocysteinaemia also causes: endothelial dysfunction, prothrombogenic activity and cardiovascular disease. 13 18 19 26 69 70
Interleukin 1-β and interleukin 6	These interleukins cause brain inflammation, particularly through microglial cells and astrocytes; DNA damage; oxidative stress; the up-regulation of glutamate resulting in excitotoxicity; and brain and systemic aging-related changes. 26 51
Parathyroid hormone (PTH)	PTH promotes mineral bone disorder, metastatic calcification, increased brain circulating and neuronal cytosol calcium levels causing changing in brain function, the induction of apoptosis due to calcium overloading, reduced regional cerebral blood flow, and somatic, behavioural and motor abnormalities. 26 59

**Table 2:** Cognitive domains affected by each of the searched UTs. The references in the table correspond to the 26 selected articles

Cognitive domains	Uric acid	Indoxyl sulphate	Homocysteine	Interleukin 1-β	Interleukin 6	PTH
Executive	X 27 28 29 33	X 34	X 35 37 38 39 40 41 42 43 Dubious: 44		X 50 52 53 54*	X 58
Attention	X 27 28		X 35 44 45		X 52 54	
Memory	X 31 35		X 37 42 43 44 45 Dubious: 35 36	X 46 47 48	X 54** 55 Dubious: 52***	
Language			X 41 43 Dubious: 35 42			
Visual-spatial	X 27 31		X 38 42 44			NA
Motor	Dubious: 28	NA	X 42 Dubious: 36 43	NA		NA

X - Significant negative associations - *p*<0.05.

Dubious - Association lost significance after adjusting for other factors.

NA - Not assessed.

PTH - Parathyroid hormone.

* Association with processing speed in cross-sectional, but not in prospective analysis.

** Association only in prospective, but not in cross-sectional analysis.

*** Association with auditory recognition memory (before correcting for demographic characteristics) but not with general memory.

## UREMIC TOXINS AND COGNITIVE DOMAINS

The categorization of cognition into discrete cognitive domains is a reductionist approach used in neurocognitive study and practice. This method allows researchers and clinicians to decompose the high-order feature called 'cognition' into less complex information processing units in order to identify patterns of impairment that can be associated with a certain disease, process, or toxin. Hence, the description of major cognitive domains - memory, executive functions, attention, language, and visual-spatial function - affected by a particular toxin can be used to establish a specific cognitive impairment pattern and identify its target areas. This can be achieved by the association between its serum levels and standardized specific neuropsychological tests.

### URIC ACID

High uric acid levels in the blood are associated with poorer attention, visual-processing speed, and cognitive flexibility in adolescent survivors of childhood acute lymphoblastic leukemia, but not in adult ones^27^. Increased uric acid in baseline levels was also associated with poorer working memory in a cohort study with cognitively healthy community-dwelling older women^28^, as well as with white matter atrophy, poorer information-processing speed, decreased executive functionality^29^ and cerebral ischemic burden^30^. It is also associated with faster cognitive decline in visual memory and visuo-construction skill in the baseline levels, although increased serum uric acid over-time was associated with a potential benefit for the attention domain and the processing speed among older men^31^. The authors of this study determined that this paradoxical situation might be attributed to uric acid antioxidant (primarily in plasma) and oxidant (primarily intracellular) function in neurons^32^. Aiming to resolve this possibility, Schretlen *et al*. performed a study that showed an association between uric acid and poor verbal and working memories, even after controlling for confounding factors^33^.

### INDOXYL SULPHATE AND P-CRESYL SULPHATE

High serum indoxyl sulphate levels are associated with a poorer executive function in the early stages of CKD, despite the lack of a significant association between *p*-cresyl sulphate and cognitive impairment^34^.

### HOMOCYSTEINE

Increased homocysteine (HCy) levels in the blood are related to greater cognitive and motor impairment, especially regarding frontal-executive function, attention^35^, verbal memory, fine motor speed^36^, processing speed, episodic memory^37^, visual, spatial and constructional ability, and processing speed^38^. High HCy levels impact negatively on task performance that assesses executive functioning^39^^,^ 40 and executive-language functioning^41^. The impact of HCy on memory is controversial: while some studies have shown none, others have reported poorer memory, motor speed, dexterity, and visuo-spatial function with higher HCy levels^42^. Executive functions and verbal expression^43^, attention, and visuoperception and construction^44^ are also impaired by HCy. In another study, HCy was found to be significantly inversely correlated with attention, and delayed but not immediate memory recall^45^. When assessing memory, the mixed results found might suggest a more specific subcomponent impairment of this core cognitive domain. There is a significant positive correlation between HCy and interleukin (IL)-6 levels^45^.

### INTERLEUKIN 1-β

There is some evidence relating IL-1β to aging processes in the hippocampus, cognitive impairment in multiple domains, and to Alzheimer disease^46^. IL-1β also impairs spatial learning and memory in animal model studies^47^^,^ 48: higher IL-1β levels hamper the consolidation processes of contextual fear conditioning and have a particular effect in the rat dorsal hippocampus^47^, which could be explained by the cytokine's interference with hippocampal long-term potentiation^49^.

### INTERLEUKIN 6

Higher IL-6 levels are associated with poorer executive function^50^, aging processes, and degeneration of GABAergic interneurons, which are essential for normal information processing, encoding, and retrieval in the hippocampus and the cortex^51^. IL-6 levels also correlate inversely with performance in tests assessing auditory recognition memory, attention/working memory, and executive function, but surprisingly not with general memory^52^. This cytokine is also associated with poorer executive function in African Americans, but not in European Americans^53^, with worse executive, attentional, and memory function, independent of cardiovascular disease and risk factors^54^, and with low performance in memory tests^55^.

### PARATHYROID HORMONE

Parathyroid hormone (PTH) crosses the blood-brain barrier^56^ and has a wide receptor distribution in the central nervous system^57^, which probably explains why alterations in calcium metabolism and, consequently, in the serum ionized calcium level (the main regulator of PTH), also impact brain function. A study has found significant negative associations between serum PTH levels and working memory capacity, and the speed of information processing^58^. A 2015 systematic review concluded that, despite mixed results, there is the suggestion of a link between PTH high serum levels and increased odds of poor cognition or dementia, although the evidence available offers weak support^59^. In this way, studies that conclusively differentiate the effects of PTH from those of the metabolites it modulates (e.g., calcium) are still necessary.

## HEMODIALYSIS AND THE COGNITIVE DOMAINS

Evidence about the effectiveness of dialysis in reducing CKD-CI is extremely relevant when considering it as a treatment at the first signals of CI. Although dialysis causes great morbidity and is a nuisance to the patient, an untreated CKD-CI for a long period will lower the patient's quality of life and may result in increased periods of hospitalization and a higher risk of mortality^26^. This is a very relevant topic because of the high prevalence of CKD-CI^8^, mainly among those older than 55 years^60^.

The association of CKD with the impairment of target cognitive domains is now being elucidated. People treated with HD have significantly lower cognitive test scores than the general population in all the domains evaluated (orientation and attention, memory, language, construction and motor function, conceptualization and reasoning, executive function, and global cognition) except for one (perception), as concluded by a systematic review and meta-analysis of 42 randomized controlled trials, and both cohort and cross-sectional studies. Tests assessing orientation and attention, memory, and executive function scored the poorest in HD patients compared to the general population. However, compared to chronic kidney disease patients not undergoing dialysis, limited evidence suggests that HD patients may perform better in memory and attention^60^.

A cohort of Dutch CKD patients performed the worst in questions that demanded memory and attention, and presented a low verbal fluency. They were born before 1979 and had started chronic renal replacement therapy at age 0-14 years between 1972 and 1992. However, this study^61^ made use of the Wechsler Adult Intelligence Scale; therefore, the cognitive domains could not be evaluated by individual tests. The authors concluded that the long duration of dialysis would enhance CI, a condition that could not be reversed even after renal transplantation, and that end-stage renal disease in childhood is associated with CKD-CI and impaired educational attainment levels in adulthood.

Starosta and colleagues^62^, in a pilot study, matched CKD patients' cognition before HD with measurements taken after the beginning of the sessions. The results suggest that HD is an effective treatment for CKD-CI, even though cognition is not fully recovered to the level of a non-CKD patient (i.e. CKD-CI persisted, but was less severe). Cognitive domains were not assessed individually in this study. In another study, Schneider and colleagues^63^ concluded that a single dialysis session (with testing performed 1 hour before and 19 hours after dialysis) improved the results in memory, attention, and executive functions. Despite the improvement, the performance of dialytic patients in post-dialysis assessments was significantly smaller than that of non-CKD patients, which highlights the lingering aspect of CKD-CI.

Interestingly, executive functions, memory, and attention are the cognitive domains most affected by the UTs that we researched in our review. This convergent pattern of cognitive domain impairments underlines the role and importance of these toxins in the genesis of CKD-CI. The fact that HD treatment ameliorates memory and attention, in comparison with non-dialyzed CKD patients, indicates that these removed UTs may affect some brain areas differently than others. We suggest that it would be beneficial for further studies to collect biochemical data about the uremic state of each patient and that cognition be assessed by domain-specific tests. In this way, specific factors may be matched more precisely with their impact on cognition, which would provide a better understanding of the mechanism of each toxic molecule and the cause of the impairment in each cognitive domain.

All these findings, together with the continual description of alterations in discrete domains, provide a finer resolution of the pattern of cognitive impairment found in uremic patients. Ultimately, these efforts have clinical significance as uremic encephalopathy needs to be distinguished from neurodegenerative diseases, delirium, cerebro-vascular diseases, and non-related psychiatric disorders, especially in a population of CKD patients, who exhibit multiple comorbidities many times.

## STRENGTHS AND LIMITATIONS

The main strength of our review is the extensive and comprehensive search on the topic of interest, which allowed us to gather a significant amount of data regarding the interaction between uremic toxins and specific cognitive domains. To the best of our knowledge, no study has reviewed such a relationship to such depth. We have also provided a strong body of evidence linking at least three UTs to three specific cognitive domains.

There are various limitations with this review: (1) the articles' biases were not systematically revised or graded after being selected for the review. Studies presenting patients with comorbidities (which make it difficult to attribute an isolated correlation between a UT and CKD) or with cognitive domain measurements made via telephone, were excluded in the selection process; (2) only articles in English were included; (3) only the PubMed database was searched; and (4) the data synthesis was not based directly on the tests used, but rather on their interpretations.

## CONCLUSIONS

Higher levels of uric acid, HCy and IL-6 are significantly associated with lower cognitive performance in executive, attentional, and memory domains. These same three cognitive domains are the most impaired in patients under HD treatment; conversely, among the cognitive domains, they present the greatest performance improvement after HD treatment, according to our literature review. This suggests a protective effect derived from the removal of uremic toxins, and highlights the important role of these three compounds in the onset of CKD-CI. In fact, when studying uremic encephalopathy, it is important to keep in mind that different uremic toxins may have different effects upon different parts of the brain, which reflects the alterations in distinct cognitive domains. This is important because it broadens the possibility of future symptomatic treatment based on the specific features a patient might present; it also helps to shed light on the biochemical background underlying each function. We hope that proper understanding of the pathophysiology of uremic encephalopathy will improve the diagnosis of cognitive impairment (which will become more clearly recognizable with the use of tests) and allow for appropriate treatment and care.

As occurred in the 20th century with cancer - a single entity that was found to be composed of a myriad of different mechanisms and types - cognitive dysfunction is bound to, in the present century, suffer the same process of deep understanding and enlightenment, since each of its facets is indeed composed of unique, distinct, underlying neural substrate. Furthermore, it is possible that each dysfunction may need a differential, mechanism-based approach in order to be tackled. With this review, we aimed to present a panorama of the intricate relationship between renal failure's uremic syndrome and the loss of full cognitive function - as both are issues to be solved per se - and to provide a window to the even most mysterious and intriguing ways of the brain.
